# Supplementary material for: Evaluation of airflow pattern and thermal behavior of the arched greenhouses with designed roof ventilation scenarios using CFD simulation
Source: PLoS One. 2020 Sep 29;15(9):e0239851. doi: 10.1371/journal.pone.0239851 (PMC7523980; doi:10.1371/journal.pone.0239851)
Supplement: S1 Nomenclature — (DOCX) [file pone.0239851.s001.docx]

| **Nomenclature** | | | |
| --- | --- | --- | --- |
| $A_{\text{V}}$ | area of the roof vent (m^2^) | $Y_{M}$ | contribution of fluctuating dilation |
| $C_{1\varepsilon}$,$C_{2\varepsilon}$ | model constants | $\alpha_{\lambda}$ | spectral absorption coefficient |
| $D_{m}$ | measured value of measurement points | $\mu_{t}$ | eddy viscosity (kg m^-1^ s^-1^) |
| $D_{S}$ | simulated value of measurement points | $\rho$ | density (kg m^-3^) |
| $E$ | flow energy (N m) | $\varepsilon$ | dissipation rate of turbulence kinetic energy (m^2^ s^-3^) |
| $G_{k}$ | generation of $k$ due to mean velocity gradients | $\lambda$ | wavelength |
| $G_{b}$ | generation of $k$ due to buoyancy | $\vec{r}$ | solar position vector |
| $\vec{g}$ | gravity acceleration (m s^-2^) | $\vec{s}$ | solar direction vector |
| $h_{j}$ | sensible enthalpy (J kg^-1^) | $\sigma_{s}$ | scattering coefficient (m^-1^) |
| $I_{\lambda}$ | radiation intensity (W m^-2^) | $\sigma$ | Stefan-Boltzmann constant (5.67×10^-8^ W m^-2^ K^-4^) |
| $J_{P}$ | microclimate inhomogeneity of the parameter (%) | $\bar{\bar{\tau}}$ | stress tensor |
| $\vec{J}_{j}$ | diffusion flux of species | $\bar{\bar{\tau}}_{eff}$ | effective viscosity shear |
| $k$ | turbulence kinetic energy (m^2^ m^-2^) | $\sigma_{k}$,$\sigma_{\varepsilon}$ | Turbulent Prandtl numbers |
| $k_{eff}$ | effective conductivity (W m^-1^ K) | $\Phi$ | diffusion phase function |
| $\bar{m}_{A}$ | average mass flow of the roof vent (m s^-2^) | $\Omega^{'}$ | solid angle (radians) |
| $n$ | refractive index | $\zeta$ | coefficient of roughness |
| $P$ | pressure (Pa) | **Abbreviations** | |
| $P_{A}$ | simulated value of the climate parameters | CFD | computational fluid dynamics |
| $P_{M}$ | mean value of the climate parameters | NMSE | normalized mean square error |
| $T$ | temperature (K or °C) | PRMSD | predicted root mean square division |
| $t$ | time (s) | VFR | ventilation flow rate |
| $U(y_{r})$ | reference wind velocity at the reference height (m s^-1^) | RS | Rolling shutter type |
| $U(y)$ | wind velocity at y height (m s^-1^) | JU | Jack-up type |
| $u_{i}$ | velocity vector in Cartesian coordinates (m s^-1^) | PW | Pivoting window type |
| $V$ | greenhouse volume (m^3^) |  |  |
